# The Risk Implications of Globalisation: An Exploratory Analysis of 105 Major Industrial Incidents (1971–2010)

**DOI:** 10.3390/ijerph13030309

**Published:** 2016-03-10

**Authors:** Matthias Beck

**Affiliations:** Queen’s Management School, Queen’s University Belfast, Riddel Hall, 185 Stranmillis Rd., Belfast BT9 5EE, Northern Ireland, UK; m.beck@qub.ac.uk; Tel.: +44-028-9097-4131

**Keywords:** industrial incidents, gobalisation, socio-political amplification of risk, risk society, neoliberalism, post-neoliberalism, developing countries, industrialising countries, developed countries

## Abstract

This paper revisits work on the socio-political amplification of risk, which predicts that those living in developing countries are exposed to greater risk than residents of developed nations. This prediction contrasts with the neoliberal expectation that market driven improvements in working conditions within industrialising/developing nations will lead to global convergence of hazard exposure levels. It also contradicts the assumption of risk society theorists that there will be an ubiquitous increase in risk exposure across the globe, which will primarily affect technically more advanced countries. Reviewing qualitative evidence on the impact of structural adjustment reforms in industrialising countries, the export of waste and hazardous waste recycling to these countries and new patterns of domestic industrialisation, the paper suggests that workers in industrialising countries continue to face far greater levels of hazard exposure than those of developed countries. This view is confirmed when a data set including 105 major multi-fatality industrial disasters from 1971 to 2000 is examined. The paper concludes that there is empirical support for the predictions of socio-political amplification of risk theory, which finds clear expression in the data in a consistent pattern of significantly greater fatality rates per industrial incident in industrialising/developing countries.

## 1. Introduction

In 1985 Barry I. Castleman published a widely read paper entitled “The Double Standard in Industrial Hazards” [1]. Castleman argued that the adverse impact of industrial activity on workers’ health was aggravated in developing countries by factors such as poor nutrition, pre-existing chronic diseases, a lack of specialised health-care expertise and climatological factors; with high temperatures facilitating the absorption of chemicals through the skin, while creating obstacles to the use of protective measures [1]. He cited a study showing that 2000 deaths from pesticides had occurred annually in the Brazilian state of Sao Paulo alone on account of problematic working practices and the illiteracy of the workforce [2]. Castleman noted that some data indicated that workers in Latin American were about 10 times as likely to suffer a workplace mishap leading to temporary or permanent invalidity than those in the UK [3]. 

One particular problem identified by Castleman was that multinational companies established in developed countries routinely exported hazardous factories to developing countries because of their lower, or non-existent, regulatory standards [1]. These included activities such as the production of textiles containing asbestos, the recovery of arsenide from copper melting and the production of carcinogenic benzedrine dyes [4,5]. In the late 1970s, for instance, Deutsche Kap-Asbestwerke dismantled its Hamburg factory and re-erected it in Cape Town, South Africa in a decision which reflected the absence of stringent regulations and strong trade unions in that country at the time [1,6]. According to Castleman, the Bhopal disaster—one of the most perilous industrial disasters of recent time—similarly reflected double-standards in the safety strategy of the parent multinational (Union Carbide), which were made possible by the absence of enforceable international standards for environmental safety [1,7]. 

Castleman’s work of the 1980s anticipated much of what later became part of the theory of socio-political amplification of risk as applied in developing country contexts [8,9]. The concept of a socio-political amplification of risk initially related to frequently observed patterns where the bulk of the cost of risks associated with hazardous technologies or production facilities were distributed unevenly either across different countries or localities or across social groups within countries, thus presenting a pattern of “risk discrimination” [10,11]. As a theoretical concept, the notion of socio-political amplification of risk can be seen as a further development of the—perhaps less useful and often misused—earlier concept of a social amplification of risk. Socio-political amplification of risk theories emphasise the impact of institutional and political frameworks in the creation of structures of vulnerability, while the social amplification literature has focused on the role of the media as well as psychological and cultural factors in amplifying public responses to risk events.

One specific version of the idea of a socio-political amplification of risk, developed in the 1990s in relation to ongoing globalisation debates, suggests that the concept relates to the “comparative powerlessness of certain societies to control risk” [9] (p. 20). This specific notion of risk amplification is grounded in the observation that technological and financial interchanges are part of an interdependent global economic system that assigns different functions in the international division of labour [9,12]. Accordingly, the international division of labour results in a concentration of consumption in industrialised countries, with about a quarter of the population consuming about 80% of global goods [9,13]. Meanwhile, the distribution of risk largely follows an opposite trajectory, with those living in developing countries being generally exposed to greater risk than residents of developed nations, primarily on account of “less elaborate measures for the protection of the environment and human health and safety” being available in less developed countries [9,13,14].

Conceptually, the idea of a socio-political amplification of risk stands in contrast to two other types of prevalent constructs about the nature of industrial risk and disaster in the modern globalised world. The first of these are neoliberal/post-neoliberal paradigms [10]. The second are theories associated with Ulrich Beck’s “risk society” hypothesis [10,15,16].

The neo-liberal literature asserting that markets will resolve social problems, including employee exposure to industrial hazards and environmental risks, is extensive and has a long pedigree. Specifically, the idea that future industrial development would ease (rather than aggravate) environmental and social problems can be traced to the 1963 work of Howard Barnett and Chandler Morse [17], which also provided one of the first broad cornucopian analyses rejecting the idea of resource scarcity. The Barnett–Morse approach was expanded upon during the 1980s in the works of Julian Simon and Herman Kahn who argued that natural resource degradation and inadequate working conditions were typical of poor societies and vanished with growth and development, while the growth of free markets simultaneously assured the development of adequate institutional frameworks for the protection of civil society [18]. Building on these views, pro-globalisation analysts such as Alan Shipman tend to associate the activities of multinational enterprises with positive risk-mitigating side-effects, arguing, for instance, that “... with big business bringing organised capability to individual action, people can acquire their public goods through comfortably private channels” [19]. Notwithstanding the lack of empirical evidence in support of this and related views, it stands to reason that even if some of these positive developments occurred, this alone would tell us little about how distributional issues were being addressed, especially in environments where inequality has historically been pronounced.

Post neo-liberal views about the nexus of risk exposure and development in developing/industrialising countries are more complex. The work of Gene Grossman and Alan Krueger, for example, suggests that the movement of industry from better regulated workplaces in the U.S. to worse regulated venues in Mexico may have some negative welfare implications, but that such costs will, over time, be vastly exceeded by benefits [20]. Specifically, Grossman and Krueger suggest that initially environmental degradation increases with economic development, but that—following an inverted U curve—later on environmental degradation per unit of economic development decreases. Added to this, it has been suggested that environmental provisions and side agreements of international treaties accelerate such processes [21].

Although Grossman and Krueger assert that Mexico has been on the threshold where further growth would sustain improvements to the environment and working conditions, evidence in relation to these claims has remained mixed [10]. This has led some commentators to argue that the presumption of “delayed gratification” in relation to the development risk exposure nexus may be conceptually problematic, as it allows for analyses highlighting negative aspects of industrial development in developing countries (and associated calls for better regulation) to be ignored at virtually any point in time, on account of the assumption that these were temporary events which would disappear once development progressed [22]. In sum, the neoliberal approach predicts a convergence in terms of incident patterns across industrialising and industrialised nations, and that such convergence should be pronounced during more recent decades, as compared to earlier periods.

In its most simple form, the risk society hypothesis, as proposed by Ulrich Beck, assumes that the distribution of poverty is “hierarchic”, in that it follows the contours of power within a society; whereas that of risks is “democratic” and universal, in that it applies to all nations or subgroups within nations equally [15,16]. Underpinning Beck’s view is the concept of mega-disasters, which he developed to describe events such as the Chernobyl or Fukushima disasters, that affected large portions of our global society—irrespective of their existing resource endowments, or levels of wealth and development. In parallel with a focus on mega-disasters and the related idea of a universality of risk exposure, risk society scholars emphasise the futility of national risk regulatory frameworks, and advocate their replacement by global regulatory frameworks. While the ideas of “risk society” and mega-disasters may have some credibility in relation to large-scale technological disasters, these views have been widely criticised on account of a number of interconnected deficiencies. These include: a lack of attention to variations in risk exposure amongst populations and workers across the globe, the failure to recognise the role of business interests in preventing necessary regulatory interventions and, perhaps most importantly, the inability to problematise the ongoing export of hazards from industrialised to industrialising countries and regions [10,22,23]. These concerns, in turn, have given rise to critiques which view risk society theories as an inadequate ethnocentric extrapolation of Western debates on technological risks and risk management into a global context, which is in realty still marked by stark inequality in wealth as well as risk exposure [22,24]. In terms of possible predictions regarding accident patterns, risk society theory would assume that large scale technical disasters are an ubiquitous phenomenon, which occur in an identical or similar form across developed and underdeveloped nations.

This paper utilises data on 105 major multi-fatality industrial disasters which took place in the periods from 1971–1980, 1981–1990, 1991–2000 and 2001–2010 to test whether there is evidence of socio-political amplification of risk in the distribution of accident patterns across industrialised as opposed to industrialising nations, or whether support can be found for the alterative hypotheses of a neoliberal convergence in hazard exposure levels or an ubiquitous increase in risk exposure across the globe. Although the data set on which this analysis is based is currently still being refined, this analysis finds support in favour of the predictions of socio-political amplification risk theory as applied to less developed nations, and this is expressed in a consistent pattern of significantly greater fatality rates per industrial incident in these regions. That said, it must be acknowledged that the analysis of historical data on disasters across global regions is always affected by data integrity issues; therefore the conclusions presented here must be interpreted with those issues in mind.

The paper is structured as follows: Section 2 discusses key ideas associated with socio-political amplification of risk theory together with recent factors which exacerbate the risk exposure of workers in developing countries. Section 3 discusses the data base underpinning the paper and explores differential levels of vulnerability in major industrial disasters across industrialising/developing and developed nations. Section 4 provides a summary and conclusion.

## 2. Global Development and Patterns of Vulnerability

According to the pioneering work of Marcelo De Souza Porto and Carlos De Freitas on socio-political risk amplification, the expectation that developing societies are more vulnerable in industrial disasters builds on a number of theoretical concepts, including models of social and environmental inequality [9,25,26,27]. Additionally, they emphasise the importance of coupling between technological and social systems that can give rise to problems in the assessment and management of risk where either system is weak or underdeveloped [9]. In combining these aspects of vulnerability, the authors predict “a higher rate of fatalities, injuries and more severe environmental destruction in industrialising countries” [9] (p. 21). They document this empirically in the context of India, Brazil and Mexico, which “have in common the adoption, in the 1960s and 1970s, of similar development models centred on rapid industrialisation” [9] (p. 21), and observe that “the search for rapid economic growth and accelerated insertion in the global economic system, led to an industrialization model that was further sustained by the absence or weakness of democratic political systems and by deep changes in the structure and organization of society” [28] (pp. 728–729). Based on World Health Organization data (WHO Commission on Health and Environment—Report of the Panel on Industry, 1992) they cite official figures of 508 deaths for the 1984 Sao Paulo (Brazil) pipeline explosion (near a shantytown), 550 deaths for the 1984 San Juanico (Mexico) LPG tankfarm explosion and 2500 deaths for the 1984 Bhopal (India) incident [9] (p. 21). 

De Souza Porto and de Freitas argue that the pursuit of this model of rapid industrialisation adversely affected all key stages of risk mitigation. At the structural phase of prevention, which relates to initial investments in plants, hazardous industries were frequently located in “highly populated or chaotically urbanised areas” [9] (p. 24), [28]. In the operational phase –when safety measures relating to routine maintenance and reviews of safety performance should have taken place—high levels of turnover, poor skill and payment levels, as well as the import of technology, militated against the safe day-to-day management of plants [9] (p. 25), [28]. Lastly, mitigant prevention, associated with post-accident medical and institutional work, was frequently undermined in developing countries by the underinvestment in health and social infrastructure and the exclusion of poor and vulnerable groups [9] (p. 25), [28].

While this analysis does not necessarily give rise to readily testable hypotheses, ideas developed in relation to socio-political risk amplification have allowed researchers to identify a number of factors which contribute to the specific vulnerability of developing countries to industrial accidents and disasters [9,28,29]. Accordingly, a number of writers, following de Souza Porto and de Freitas, have tended to include the following in their causation of the added vulnerability of developing countries [9,28]: (a) an international division of labour which allows for the export of hazards and the existence of double standards in issues of environmental safety and worker protection [1,5,9,13,14]; (b) a prioritisation of growth by the host nation fostering models of rapid industrialisation that are sustained by rapid social change and the absence (or weakness) of democratic institutions [9]; (c) rapid urbanisation which includes the settlement of formerly rural poor in areas vulnerable to pollution, spills, flooding, environmental degradation and industrial accidents [7,9,30,31]; (d) strong elites with vested interests in rapid growth who are actively opposed to potentially costly risk-mitigating interventions (ranging from land-use and emergency planning to the provision of information to communities and workers) [7,9,32,33,34]; and (e) the absence of strong trade union representation which reflects the multinational company’s choice of location as well as the politics of the host nation [1,9]. Again, anticipating some of this analysis, Castleman’s early work cites the example of the South African Machinery and Occupational Safety Act of 1983, which allowed employers to appoint worker representatives, and frequently led to the creation of pseudo-consultative committees [1].

These views are echoed by other researchers, such as the disaster analyst Enrico Quarantelli, who investigated urban vulnerability to disasters in developing countries [29]. Like de Souza and de Freitas, Quarantelli highlights the particular challenges posed by poverty and poor housing, by quoting Bowonder and Kasperson’s statement that “people who are already barely eking out an existence will not avoid a risky flood plain or the shadow of a volcano any more than they will eschew the squatter settlements around a pesticide factory in Bhopal or a liquefied gas facility in Mexico City. In short, the poorest of the poor are probably likely to reside in the path of both natural and technological hazards” [29] (p. 212) citing [35] (p. 104). To this he adds the observation that many developing countries such as Thailand—where industry is heavily concentrated in the Bangkok area—have experienced a strong concentration of industrial activities in relatively small areas, with hazardous industrial activities frequently being located in low income areas whose residents are “less able to … deal with threats or respond to, crises” [29] (p. 212). Quarantelli suggests that “when technological and natural disasters strike these urban areas, the human and social costs are greater” because these populations are “already less protected from the elements and struggling for daily survival” [29] (p. 213). Where informal settlements are considered illegal, they will usually not be included in land use and emergency plans [29]. Other factors contributing to social vulnerability encompass the unusually large numbers of disabled and otherwise vulnerable individuals that can be found in post conflict areas as well as areas ravaged by diseases such as AIDS [29] (p. 214). Water shortages and droughts—such as have been experienced repeatedly in Sao Paulo—together with water and air pollution, and soil erosion can further increase the vulnerability of urban populations in developing countries to technological and natural disasters [29] (p. 215). Added to this are the frequently observed vulnerability of the transport and communications infrastructure and the lack of regulatory oversight relating to hazardous material transport, factory machinery, work premises and storage facilities. In many cases these problems are further aggravated by the virtual absence of disaster and emergency planning which is often rooted in a lack of resources rather than a lack of awareness of risks [36]. These problems can be particularly pronounced in developing countries where multinational investors negotiate with central governments, but are less likely to take into account the views of local communities about where facilities will be sited [29].

While much of the literature on the socio-political amplification of risk in developing countries written in the two decades to 2000 has focused on the role played by multinational investors, more recent studies have added three new themes to this debate. These are: structural adjustment reforms and their effects on the workforce of industrialising countries, exports of waste and hazardous waste recycling and new patterns of domestic industrialisation. 

### 2.1. Structural Adjustment Reforms

Researchers have tended to explore structural adjustment reforms in conjunction with frequently parallel developments of trade liberalisation and globalisation more generally [10]. In this context, it has been argued that structural adjustment reforms, in particular, have reduced states’ “right/ability (or ‘infrastructural power’) to regulate the domestic market, the environment and the health and safety of workers” at the very time when newly democratised regimes created a demand for such regulation [10] (p. 328), [37]. That said, it is worth noting that international organisations such as the World Bank—as drivers of structural adjustment—have undergone changes *vis-a-vis* questions of workplace health and safety. Thus, early statements by World Bank leaders, which seem to have been reversed later on, seem to indicate a shockingly casual attitude to the dangers associated with the export of industrial hazards. These early casual attitudes can be illustrated by a 1991 memo written by then chief economist of the World Bank, Lawrence Summers, in which he stated that [10] (p. 334) citing [38] (p. 66):

(1) The measurement of the costs of health-impairing pollution depends on the forgone earnings from increased morbidity and mortality. From this point of view a given amount of health-impairing pollution should be done in the country with the lowest cost, which will be the country with the lowest wages.
(2) The costs of pollution are likely to be non-linear as the initial increments of pollution probably have been very low cost. I’ve always thought that under-polluted countries in Africa are vastly under-polluted; their air quality is probably … low compared to Los Angeles or Mexico City…
(3) The demand for a clean environment for aesthetic and health reasons is likely to have very high income-elasticity. The concern over an agent that causes a one-in-a-million chance in the odds of prostate cancer is obviously going to be much higher in a country where people survive to get prostate cancer than in a country where under-5 mortality is 200 per thousand. Also, much of the concern over industrial atmosphere discharge is about visibility of particulates. These discharges may have little direct health impact. Clearly trade in goods that embody aesthetic pollution concerns could be welfare enhancing.

Some observers have argued that Summers’ memo reflected much of the then developing new *status quo* with regard to the export of hazardous industries to developing countries [10]. In this context, it was suggested that Summers’ view described a growing economic as well as political reality which had evolved from the interaction of economic globalisation with political demands for structural adjustment policies. Accordingly, the geographer David Harvey commented that “if areas where low-income, disempowered, and marginalized ‘others’ live are also zones of ... weak political resistance, then the symbolic, political, and economic logic for the location of noxious facilities works in exactly the way that Summers’ memo envisages” [10] (p. 335) citing [39] (p. 368).

Over a period from about 1996 to 2004 a number of studies have documented the adverse effects of structural adjustment policies on employment levels, job security, real wage levels, income distribution, worker rights and trade union participation in countries, such as Ecuador, El Salvador, Mexico and Zimbabwe [40,41,42,43]. The World Bank responded to such criticism by adopting, in 2006, a series of new standards for private sector companies [40]. These standards were intended to guide the activities of the International Finance Corporation (IFC)—a World Bank organisation—providing loans and guarantees to private sector companies domiciled in, or investing in, developing countries [40]. They require, among other stipulations, that businesses involved with IFC financed projects meet core labour standards as formulated by the International Labour Organisation (ILO) [40].

However, despite claims of improvements, detailed case studies of specific programmes such as the UN- and ILO-sponsored Better Factories Programme Cambodia (BFC), which is meant to monitor garment factories, suggest that such interventions can be completely ineffective [44]. Real wages in the Cambodian garment sector have fallen by 16.6% over the past decade. In 2012 “86% of factories monitored in a six-month period did not respect the legal daily overtime limit, while 62% did not provide sufficient ventilation” [44] (p. 1) citing [45]. Apart from an increased usage of child labour, it was also observed that factory managers used overtime to “to keep the overall number of workers they employ lower, thus reducing their per-worker overhead costs accordingly (such as the obligation to grant workers attendance bonuses, seniority bonuses, severance pay, and maternity benefits)” [45] (p. 11).

### 2.2. Exports of Waste and the Rise of Hazardous Waste Recycling

One of the more recent developments to exacerbate the risk exposure of workers in developing countries is the export of waste and the rise of hazardous recycling industries. One of the first large-scale hazardous industries to move to developing countries has been ship-breaking [46]. Ship-breaking first came to play a major role along the Gadani coastline of Pakistan from the 1970s onward. Gadani declined, as Bangladeshi and Indian ports started establishing competing enterprises. International observers have highlighted the dangers posed to workers by this industry on account of the presence—especially among older vessels—of carcinogens and poisons such as asbestos, lead, polychlorinated biphenyls and heavy metals, as well as risks associated with fire, explosions, suffocation and mutilation from falling metal; and it has been suggested that, because of the presence of such highly dangerous substances and the cost associated with their safe disposal, ship-breaking in most developed countries is no longer economically viable [47]. 

The issue of the export of asbestos hazards and ship-breaking came to global attention through the Indian Supreme Court’s 2007 decision to allow the dismantling of the ship Blue Lady in Alang [46]. In 2007 Alang, in the state of Gujarat, was the largest ship-breaking yard in the world and employed about 40,000 workers. Following a downturn in activity, due to local requirements for certificates stating that oil tankers were free of gas residue and a dispute over the planned breakup of the French aircraft carrier Clemenceau, the acquisition of the Ocean Liner Blue Lady by an Indian breaking company was seen as a chance to revive the industry. Anchored in Bremerhaven in 2004, it had been originally decided that, due to the large amounts of asbestos contained in the ship (then named The Norway), it would not be allowed to leave the country for dismantling in line with the Basel Convention. The ship eventually was moved to Malaysia in 2005 under the pretence of repairs and then sold to a Liberian shipping company which named it Blue Lady. Following claims that the ship would head toward the United Arab Emirates, it became clear that the ship was in fact being moved to Alang—leading the environmentalist and an anti-asbestos activist Gopal Krishna to file a lawsuit in the Indian Supreme court with the support of a number of NGOs. After some delays the court declared the ship safe to be scrapped in a much criticised decision. The principle rationale of the court was that, notwithstanding danger associated with asbestos and other chemicals, the break-up provided tangible benefits to the Indian economy such as “41,000 MT steel” and “employment for 700 workmen” [46] (p. 141). In this context, the court further noted that “India after globalisation is an emergent economy along with Brazil, Russia, and China with an economic growth of above nine per cent. However, that growth is lop-sided. A large section of the population lives below the poverty line. Unemployment is endemic in India” [46] (p. 141). There is little doubt that the court’s assessment of the economic realities is correct. However, it stands to reason that the absence of appropriate controls will result in hardship to poor workers in particular, while the reluctance to support appropriate regulations will likely result in a regional race to the bottom where different jurisdictions offer concessions to industries which would not be sufficiently profitable were they to pay for the necessary protective measures.

The close link between predictions of socio-political amplification of risk theories and hazard creation in these industries can be gleaned from the summary of a 2008 study of occupational hazards facing ship scrapping workers at Chittagong Coastal Zone [47] (p. 141):

“Workers break the obsolete vessels with no protection from explosions, infiltration of asbestos, heavy metals, oil residues, TBT, PCBs, or a cocktail of toxic chemicals contained in the ship. Most of the workers are not aware of the ship borne poisons and their impacts on health and thus they continue work without any protective measures. ... The main causes of accidents in ship scrapping yards are due to sudden fall of steel beams, burning and detonation of gas, suffocation by inhaling CO_2_ and other obnoxious gases trapped in ships’ chambers. Deep cut; burning; breaking and fracture of bones of hands, legs, fingers and head; fainting and loss of limb are the most common accidents. Most of the workers were found to suffer from multiple diseases and health hazards. Poor safety systems, hazardous working conditions, use of traditional methods of cutting giant ships, absence of appropriate emergency response and lack of precautionary measures are the main reasons for accidents and casualties. There is no health care facility in the surroundings of the ship scrapping zone. Thus, in case of accidents, the patients have to be rushed to the City centers about 10–15 km away, where adequate medical facilities are available. But distance, traffic congestions and high cost are the main hindrance to avail those facilities by the low waged labourers.”

Another hazardous recycling industry is what is called the e-waste industry. E-waste includes discarded computers, monitors, televisions, cell phones and many other electronic products [48,49]. Estimates indicate that between 30 and 50 million tons of e-waste are discarded annually and exported to China, Bangladesh, India, Malaysia, Pakistan, the Philippines, Vietnam, Ghana and Nigeria [48] (p. 81). For the U.S. alone, it has been estimated that 80% of e-waste is exported with 90% of that waste going to China [48,50]. Frey notes that, as one of the largest e-waste recycling sites in the world, Guiyu Township in China employs 150,000 e-waste workers, including children and commuters [48] (p. 61). He highlights that “hazardous materials include heavy metals, brominated flame retardants, and many other toxic materials; lead and cadmium and mercury in circuit boards; lead oxide in CRTs; mercury in switches and flat screen monitors; cadmium in computer batteries; and persistent organic pollutants (dioxins, PVCs, and PAHs) in plastics” [48] (p. 82) citing [51,52]. The extraction of material involves the breaking of cathode tubes with hammers which releases toxic phosphor dust (with copper yokes being sold to metal dealers); the cooking of circuit board over open fires to melt lead solder (which again is sold to metal dealers); the melting of plastic into rods or granules (to be sold to plastic manufacturers); and the use of acid baths to extract precious metals [48] (p. 82). Residual plastic casings, meanwhile, are burnt while leaded glass pieces, acids and dissolved heavy metals are dumped, often into local waterways [48] (p. 82).

Although e-waste recycling is unlikely to result in large multi-fatality industrial disasters associated with major industrial enterprises, this manifestation of global industrialisation considerably exacerbates the health risks faced by workers in developing countries, as well as creating new patterns of environmental pollution which predominantly affect poor and vulnerable populations.

### 2.3. New Patterns of Domestic Industrialisation

Major industrial accidents can occur during processing, storage and transport of hazardous materials, in ways which put workers and communities at risk in the short run and also over long periods by exposing individuals to persistent toxins and latent disease causes [53] (p. 103). A recent publication by the United Nations Environment Programme cites a report by the Association for Sustainable & Responsible Investment in Asia (ASrIA) which notes that the supply chain from Asia to Europe and North America “is brittle and unprepared to address many of the emerging toxic chemical issues” [53] (p. 103) citing [54]. According to ASrIA, these problems arise from too much “permissive regulation and the fact that safer supply chains are more expensive and therefore can be undercut by lower cost producers” [53] (p. 103). One particular problem identified in this context is the prevalence of mislabelled bulk chemicals in the Chinese market, which arises from the fact that suppliers substitute locally available cheaper sub-standard chemicals for international standard products. Compliance with health and safety or environmental standards often means that there is “one compliant manufacturing line in a factory with multiple lines” [53] (p. 103). These practices are implicitly encouraged where consumer electronics manufacturers will not “commit to a new safer component ... that cannot be second-sourced from a competing supplier” [53] (p. 103). 

The potential risks associated with these practices are aggravated on a local level by the particular development patterns that have characterised the chemical industry in developing countries in Asia. Accordingly, the 2013 UNDEP publication, *Global Chemicals Outlook*, cites the China Petroleum and Chemical Industry Federation (CPCIF) as reporting “that there are 189 main provincial chemical parks and industrial parks across 25 provinces, municipalities and autonomous regions in China (except Guangdong, Guangxi, Qinghai, Xizang, Anhui and Guizhou) (with)...more than 1000 chemical parks and chemical clusters existing or under construction” [53] (p. 106). Of these new parks, “less than 100 ... have been planned or constructed integrating the necessary management capabilities on safety and environmental protection” thus increasing the “probability of ‘domino-effect’ accidents due to the large quantity and variety of chemicals and hazardous installations in close proximity to one another” [53] (p. 106). Evidence of a significant number of major incidents in the Chinese chemical industry is widespread. In March 2005, a road tanker carrying toxic liquid overturned in Huai’an, killing 28 people who inhaled the poisonous fumes that escaped, and in April 2005 an explosion at Dongxi chemical plant in Chongqing resulted in 12 deaths [55] (p. 4). In July 2006, a blast in a fluorination reactor which had been operating without government authorization at Yancheng Fuyuan Chemicals in Jiangsu province killed 22, while an explosion at Dangtu Longsheng Chemicals in Anhui province killed 16 workers [55] (p. 2).

As exemplified by the development of the chemical industry in China, there are indications that new patterns of domestic industrialisation encounter many of the problems highlighted by Quarantelli in relation to the concentration of industrial infrastructure near urban areas and poor planning more generally, despite the fact that their development no longer involves multinationals trying to seek the extraction of favourable conditions from governments [29]. Some of this seems to be attributable to what could be loosely described as the dynamics of a “race to the bottom” between regions seeking to attract new domestic investment. A similar phenomenon has been observed with regard to the erosion of environmental regulation among U.S. states competing for investment [56,57,58] and, on a global scale, in relation to labour standards [59]. Other causes may stem from the fact that countries like China have only partially modified the pro-industrialisation bias which de Souza Porto and de Freitas identified in connection with India, Brazil and Mexico for the period from the 1960s to the 1990s [28]. As matters stand, the combination of structural adjustment reforms, the export of waste and the rise of hazardous waste recycling, as well as problematic patterns of domestic industrialisation in developing countries are all likely to intensify the patterns of socio-political amplification of risk highlighted by earlier research [1,5,6,7,8,9,28,29].

## 3. Data Analysis

As noted above, the core implication of the application of socio-political amplification of risk theory to developing country contexts is the prediction that societies in developing nations will be more vulnerable to industrial disasters than those in developed countries. This vulnerability of industrialising/developing societies can find its expression in a number of measurable outcomes, including: a greater absolute or relative incidence of large-scale or multi-fatality disasters in these countries, a wider or more severe impact on adjacent populations and a greater number of those who are fatally injured per incident. It would also be reflected in higher counts, and a greater burden, of occupational illness in developing countries, but this has rarely been investigated quantitatively on account of the severe patterns of underreporting.

Events generally tend to confirm the predictions by socio-political amplification of risk theorists of a measurably greater incidence of fatalities occurring in individual industrial disasters of developing countries [8,9], as well as the related predictions of disaster management experts such as Quarantelli [29]. The trend towards greater loss of life in industrial disasters has been observed specifically for India, Brazil and Mexico in studies of the late 1990s and early 2000s [9,28], and, more recently, in an important 2012 study of industrial disasters by Efthimia Mihailidou, Konstantinos Antioniadis and Marc Assael [60]. A 2008 study focusing on energy related accidents did not confirm the trend, finding instead a concentration of disasters in the USA [61]. However, the author of that study conceded that this may have been due to data bias, since: “the USA and its territories consume only around two-fifths of the world’s oil and one-quarter of its coal and natural gas, and are home to only 4.5 percent of the world population” which makes it “extremely unlikely that the country is actually home to more than 70 percent of all energy accidents” and “more likely that better media coverage, and the fact that sources are in English, are behind the prevalence of American energy accidents” [61] (p. 1809).

For the purpose of this analysis, emphasis has been placed on issues of data integrity in comparing the incidence of fatalities in developing and developed nations in recognition of the different propensity of countries to report industrial incidents and/or the scale of their effect. Accurate reporting of fatalities can be affected by such factors as the density and coverage of media reporting in a country or region, government interference with media, potentially differing cultural sensitivities with regard to adverse events and their effects, company practices with regard to the openness of reporting, as well as regulatory requirements and levels of regulatory oversight [62,63]. As a consequence of these divergences, comparisons of reported data in relation to minor or major injuries across developing and developed countries generally have to be considered problematic (as the distinction of minor and major injuries is subject to local definitions, or is unavailable as it requires a level of medical investigation and sophistication which may not be present at a specific accident location). Even where the number of injured persons is reported as a single category, inaccuracies can arise from poor diagnosis due to low levels of medical provision at, or near, the accident site, or the unwillingness of individuals to report their injuries where they would be liable to pay for medical attention (and/or compensation is unlikely). Lastly, there can be a lack of interest in reporting “lesser” industrial accidents where unnatural deaths are a frequent occurrence (the Mexican drug wars, for instance, are said to have resulted in over 100,000 deaths in a period of six years). Similar considerations apply to the reported number of persons evacuated after an industrial disaster. This is likely to depend on the willingness of company operators or authorities to initiate an evacuation, while political, regulatory and cultural factors may affect compliance levels. This was made painfully clear in the Bhopal disaster where the failure of company operators Union Carbide to stress the urgent need for evacuation contributed to the death toll of this disaster [7].

This study takes as its start-out point the aforementioned 2012 study by Mihailidou *et al.* [60]. Some of the advantages of this important work are, firstly, that the authors applied a detailed and consistent definition of what to include under the category of a major industrial accident and, secondly, that they identified the source of information which they used for each accident entry. In order to reduce the potential for misleading data comparisons a number of further modifications and augmentatiosn where conducted to the Mihailidou *et al.* data set. First, the paper presented here includes in its data base only those entries of the Mihailidou *et al.* paper, which occurred after the year 1970. This decision was based on the fact that earlier entries were far more likely to be affected by reporting biases, than those of later years (due to the global expansion of media and news coverage). This reduced the number of entries from 319 incidents reported by Mihailidou *et al.* for the years from 1917 to 2011 to 271 for the period from 1971 onwards. A second measure aimed at strengthening data integrity and comparability was to require a threshold of a minimum of five deaths in addition to the other criteria imposed by Mihailidou *et al.* This was based on the assumption that there was some possibility that incidents occurring especially in developing countries with fatalities falling below that figure would not attract the attention of national or international government organisations such as UNEP, or that of widely disseminated mainstream media. This further reduced the number of entries drawn from the Mihailidou *et al.* data base from 271 to 92. Following this initial data reduction, other databases and sources were investigated for any incidents which would meet the criteria of the Mihailidou *et al.* data base, plus the minimum five deaths or more requirement. One of these databases was Benjamin Sovacool’s 2008 paper “The Costs of Failure: A Preliminary Assessment of Major Energy Accidents, 1907–2007” which lists a total of 281 accidents which occurred in the energy sector from 1923 to 2007 [61] (pp. 1810–1819). Applying the aforementioned criteria, this yielded an additional 10 incidents for the relevant period. These included, among others, the 1975 failure of the dam at the Shimantan hydroelectric facility, Henan Province, China, which caused 26,000 immediate deaths with another 145,000 persons succumbing to their injuries during the subsequent epidemic and famine [61]. A further three accidents were added to the database on the basis of information gleaned from reports created by Independent Chemical Information Services [55]—bringing the total number of accidents included to 105. Where one source provided information that appeared more accurate than another, these details were substituted in the table. Overall it can be assumed that the combination of the relative up to date Mihailidou *et al.* data base with the Sovacool analysis and data derived from Chemical Information Services provides a relatively consistent and reliable underpinning to this analysis which in this sense should match or exceed comparable older data sets [64,65,66,67,68,69,70]. Despite these measures, there is no guarantee that all disasters that would warrant inclusion are indeed included, and in many ways this work must be considered a best estimate. This applies in particular to incidents which occurred in the early two decades of this data base. One example of this is the 1993 Shenzen series of explosions which apparently killed an estimated 12 workers and injured 168, but became known to Western media mostly because fires could be seen from Hong Kong [71]. 

The tables below were produced using a combined database of major industrial accidents which includes a total of 105 such events that occurred from the beginning of the year 1971 to the end of the year 2010. For ease of display, the data are presented in four blocks (1971–1980, 1981–1990, 1991–2000 and 2001–2010) with summary data displayed at the end of each table. The data listed in the tables include: (i) the date of the event (modified/checked from: [55,60,61]); (ii) country and location (modified/checked from: [55,60,61]); (iii) number of injured rounded to the nearest “10” (modified from [55,60]); (iv) fatalilities (modified/checked from [55,60,61]; (v) evacuations rounded to the nearest “100” (modified from [60]); (vi) context (modified from [55,60,61]); (vii) estimated cost (in million 2010 USD: with constant 2010 Euro prices from [60] being transformed into 2010 USD prices at *1 Euro (2010) = 0.75 USD (2010)*; and constant 2007 USD figures from [61] being transformed to 2010 USD figures at *1 USD (2007) = 1.07 USD (2010)*; (viii) a classification of the country as industrialising (I) or developed (D); and (ix) source from which the information has been derived. Data are shown in descending chronological order. The classification of countries into the “industrialising” or “developing” category is similar to that employed by Mihaildou *et al.* who utilise a 2007 IMF survey for this purpose [60] (p. 536). In part following this approach, a joint measure of GDP size and share of workers employed in industry was utilised. This measure ensures that countries that are at the early stages of industrialisation and do not produce high levels of industrial output are classed as “industrialising” (e.g., India) in line with the emphasis of socio-political amplification of risk theory on regulatory and infrastructural development (which typically accompany the later stages of industrial development). It also allows for relatively wealthy countries with low levels of overall industrial development (such as Quatar) to be excluded from the “developed” category and classed as “industrialising”—which again would reflect their frequently low levels of regulatory and infrastructural development. 

### 3.1. Period A, 2001–2010

For the most recent period, a total of 18 major industrial accidents satisfied the criteria for inclusion in the database (see Table 1). The most severe was the explosion of an oil pipeline on 26 December 2006 in Lagos, Nigeria which caused widespread fires that destroyed more than 300 homes and killed 466 persons [61] (p. 1819). A total of 5 major disasters occurred in developed countries *(c1)* while the remaining 13 industrial disasters *(e1)* occurred in industrialising countries. The average number of fatalities occurring per incident in developed countries was significantly lower than in industrialising countries, with 24.0 deaths per accident (*d3)* as compared to an average of 107.1 *(f3)*, respectively. This matches the prediction of socio-political amplification of risk theory which assumes that the severity of industrial disasters in terms of fatalities will be greater in developing/industrialising countries. The ratio of injuries to fatalities, meanwhile, was greater for developed nations with an injury-to-death ratio of 27.5 *(c5)*, as compared to industrialising nations where it stood at 3.5 *(e5)*. This again is supportive of socio-political amplification theory that anticipates a greater adverse effect of incidents in developing contexts as well as a lower propensity of injuries being fully reported in industrialising countries.

### 3.2. Period B, 1991–2000

For the period from 1991 to 2000, a total of 29 *(a1)* major industrial accidents satisfied the criteria to be included in the data base (see Table 2). The most severe of these was again an oil-related incident occurring in Nigeria. This involved the rupture and explosion of a gasoline pipeline on 17 October 1998 in the Niger Delta which destroyed two villages, killing 1078 persons. On the whole, a total of eight incidents occurred in developed countries *(c1)* while the remaining 21 industrial disasters *(e1)* occurred in industrialising countries. The average number of fatalities occurring per incident in developed countries was again significantly lower than in industrialising countries, with 27.1 deaths per accident *(d3)* as compared to an average of 139.0 *(f3)* in industrialising countries. This again matches the prediction of socio-political amplification of risk theory which would expect the severity of industrial disasters in terms of fatalities to be greater in industrialising countries.

### 3.3. Period C, 1981–1990

For the period from 1981 to 1990, a total of 28 *(a1)* major industrial accidents satisfied the criteria to be included in the data base (see Table 3). Overall, a total of 11 incidents occurred in developed countries in the period *(c1)* while the remaining 17 industrial incidents *(e1)* occurred in industrialising countries. These included two exceptionally severe accidents. Firstly, the release of Methyl Isocyanate on 3 December 1984 at the Union Carbide Chemical plant which led to the estimated deaths of 20,000 people and caused injury to about half a million others living near the plant. Some of the environmental problems associated with the Bhopal tragedy are ongoing, particularly concerning ground water and other forms of environmental pollution, which continue to blight the lives of residents of the area. On 26 April 1986, the mishandling of a reactor safety test at Chernobyl nuclear reactor caused an explosion and meltdown, necessitating the evacuation of 300,000 people from the Kiev area [61] (p. 1816). It has been suggested that the accident at Chernobyl released more than 100 times the radiation of the atom bombs dropped on Japan and that this fallout is concentrated in areas of Belarus, Ukraine and Western Russia. Despite these far-reaching effects, the International Atomic Energy Agency has estimated that only 4056 deaths resulted from the Chernobyl nuclear accident [61,72]. This figure has been included in this study for purposes of consistency, although other studies put the numbers above 200,000 and 350,000 [61] (p. 1804). Both Bhopal and Chernobyl in this sense highlight the limitation of attempts to quantitatively assess levels of harm, especially where these arise from mega-disasters with complex and long-term consequences.

Partly due to the impact of these two events, comparative figures on the average fatality rate associated with major industrial incidents in developed and developing countries for the period 1981–1990 differ more markedly than they did for the previous two periods. The average number of fatalities occurring per incident in developed countries was dramatically lower than in industrialising countries, with 37.4 deaths per accident *(d3)* as compared to an average of 1552.3 *(f3)* in industrialising/developing countries. This represents perhaps an extreme confirmation of the predictions of socio-political amplification of risk theory in terms of a massive deviation of fatality rates associated with major industrial accidents in industrialising countries from the norm of developed countries. Interestingly, for this period the rate of injuries to fatalities in developing countries exceeds that recorded for developed countries, which may be due to the fact that the obvious widespread impact of the Bhopal and Chernobyl disasters will have made it impossible to ignore the high rates of injuries associated with these events.

### 3.4. Period D, 1971–1980

For the final period from 1971 to 1980, a total of 30 *(a1)* major industrial accidents satisfied the criteria to be included in the data base (see Table 4). The most severe of these was the collapse on 8 August 1975 of the Shimantan Dam in China’s Henan Province, which had been built in the 1950s as a Soviet style hydroelectric facility. Following a Typhoon which caused massive rainfall, the reservoir filled to more than twice its capacity, with sluice gates being clogged by sedimentation [61,73]. When the dam failed, 16 billion tons of water escaped, creating a six mile wide flood-wave which destroyed 4600 square miles of property [61,74].

As concerns the distribution of major industrial accidents for this period, a total of 20 incidents occurred in developed countries *(c1)* while the remaining 10 industrial disasters *(e1)* occurred in industrialising countries. This may, in part, be a reflection of limited reporting of such incidents for developing countries during this earlier period. The average number of fatalities occurring per incident in developed countries was dramatically lower than in industrialising countries, with 37.7 deaths per accident *(d3)* as compared to an average of 17,141.4 *(f3)* deaths per accident in industrialising/developing countries. As in the case of the previous period, this again represents perhaps an extreme case of confirmation of the prediction of socio-political amplification of risk theory in terms of a massive deviation of fatality rates associated with major industrial disasters in industrialising countries from the norm of developed countries.

Overall, the notable prevalence of incidents being reported in this period for the U.S. would indicate the possibility that cross-country or regional comparisons for the years before 1980 may be limited by the accuracy and reliability of available English language reports. While international efforts have been made to provide accident and disaster data on a global basis, these generally tend to cover only a maximum of approximately the last three decades, and data reaching back further probably has to be viewed with great caution.

## 4. Conclusions

The foregoing analysis has identified significantly higher average numbers of fatalities occurring in major industrial accidents in industrialising/developing countries as compared to developed countries for the four periods of 1971–1980, 1981–1990, 1991–2000 and 2001–2010. For the most recent—and arguably most reliable period of 2001–2010—the average number of fatalities per major incident in industrialising/developing countries is 107.1 as compared to 24.0 in developed countries. For the period from 1991 to 2000, the figures follow a broadly similar pattern with an average number of fatalities per major incident in industrialising/developing countries of 139.0 as compared to a rate of 27.1 for developed countries. For the earlier period of 1981–1990, the pattern is even more pronounced, primarily on account of two mega-disasters occurring in industrialising/developing countries (Bhopal and Chernobyl). Specifically, the average number of fatalities in industrialising/developing countries in this period is 1552.3 per accident as compared to 37.4 for developed countries. A similar pattern is apparent for the earliest period of 1971–1980 which is also marked by the occurrence of a technical mega-disaster—the collapse of a dam in Henan Province, China. For this period the average number of fatalities in industrialising/developing countries is 17,141.4 fatalities per accident as compared to 37.7 for developed countries (albeit that this is mostly driven by the collapse of the Henan Province Dam).

**Figure 1:**
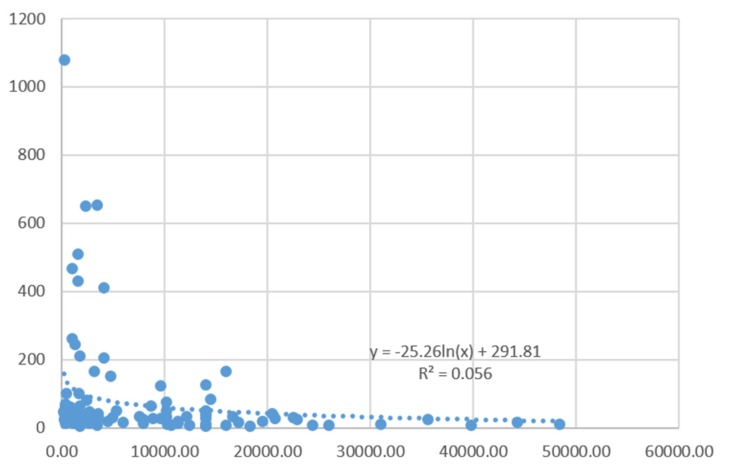
Fatalities and Country GDP per capita—Linear Scales (outliers excluded).

**Figure 2:**
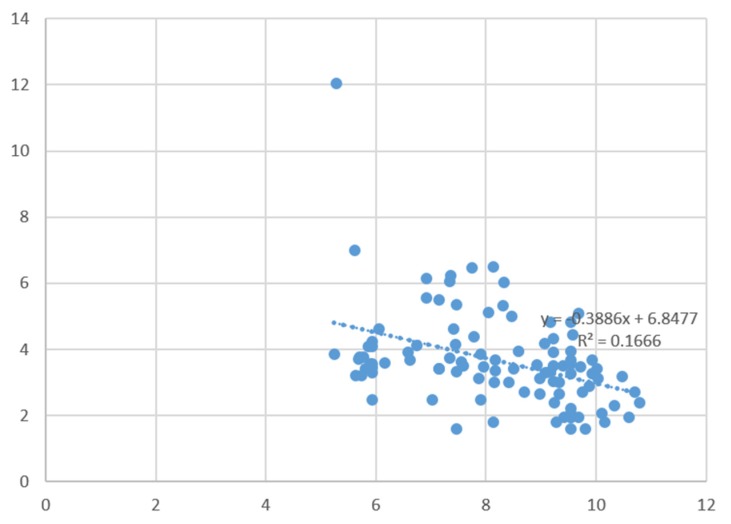
Fatalities and Country GDP per capita—Log Scales (outliers included).

Issues of data integrity and outliers (mega-disasters) notwithstanding, there is therefore a strong indication that accident patterns in developed and developing countries continue to follow the predictions of socio-political amplification of risk theorists, with workers in developing/industrialising countries suffering far greater harm than those in more prosperous developed countries. This pattern can be further illustrated by looking at the relationship of fatalities and wealth indicators for the countries in which they occurred. Figure 1 and Figure 2 depict these relationships. As can be seen from Figure 1, which displays the number of fatalities on the y axis (with outliers Bhopal, Chernobyl and the Henan dam collapse removed), and country GDP per capita on the x axis, these data follow a negative exponential trend with severe multi-fatality accidents being concentrated among poor countries and becoming rarer with high GDP per capita levels (see Figure 1).

These data can be further simplified by transforming both axes through natural logarithms in order to reduce large differences in scale on both the fatalities and GDP axes (which allows for the inclusion of previously removed outliers). As shown in Figure 2, this transformation makes visible a strong negative correlation between the severity of major industrial accidents as measured by the number of fatalities occurring per accident and the wealth of a country (measured by the country GDP per capita), with 16.6% of the total variation in the number of fatalities being explained by country wealth alone (see Figure 2).

There is no conclusive proof as to whether the predictions of socio-political amplification of risk theorists hold true in various different contexts, and the very complexity of industrial incidents would make causation difficult to prove. However, there is a strong indication that this family of theories performs better than alternative constructs as predictors of the nature and distribution of risk exposure of workers across the globe. This applies to both neoliberal predictions of a swift convergence of working conditions in industrialising/developing countries with those of developed countries, as well as risk society related assumptions about a global increase in risk exposures.

As a final note to this data analysis it is worth pointing out that it is very difficult to identify any trends in data for the four periods in question. For instance, average fatality rates for developed countries decreased moderately from 27.1 fatalities per major accident in 1991–2000 to 24.0 for the period from 2001 to 2010, though this does not constitute a statistically significant change. Fatality rates for industrialising/developing countries, meanwhile, seem to be driven by the occurrence of mega-disasters (Bhopal, Chernobyl and the Henan dam collapse) for the early periods of 1971–1980 and 1981–1990. For the two more recent periods, again, a moderate improvement can be detected, with an average fatality rate of 139.0 for the period 1991–2000 and 107.1 for the period 2001–2010; but, again, this is not statistically significant. This absence of statistically significant trends toward improvement perhaps should not be surprising, given the new forms of hazardous industries and practices that have evolved in industrialising/developing countries.

## Figures and Tables

**Table 1:** Period A, 2001–2010.

**Date**	**Country/Location**	**Inj.**	**Fatal.**	**Evac.**	**Context**	**Cost**	**Type**	**Source**
16/08/2010	China Harbin City	150	20	2000	Explsvs Plant		I	[60]
20/04/2010	USA Gulf of Mexico		11	100	Oil Drilling	10,845	D	[60]
29/10/2009	India Jaipur	150	12	500,000	Oil Depot		I	[60]
26/08/2008	China Guangxi Zhuang	60	20	11,500	Adhesive Plnt		I	[60]
25/03/2008	Iran Arak	40	30		Detergent Plnt		I	[60]
07/01/2008	South Korea Icheon	10	40		Chem.Warehs		D	[60]
26/12/2006	Nigeria Lagos City		466		Oil Pipeline	81	I	[61]
12/05/2006	Nigeria Lagos City	20	260		Oil Pipeline	25	I	[60,61]
19/02/2006	Mexico San Juan de Sabinas		65		Coal Mine/Gas	5	I	[61]
13/11/2005	China Jilin	60	5	10,000	Petrochm Plnt		I	[60]
29/03/2005	China Huai’an Jiangsu	350	28		Chlorine Spill		I	[55]
23/03/2005	U.S. Texas City	170	15		Oil Refinery	149	D	[60]
14/02/2005	China Fuxin		210		Coal Mine/Gas	13	I	[61]
30/07/2004	Belgium Ghislenghien	130	24		Gas Pipeline	15	D	[60,61]
20/01/2004	Algeria Skikda	70	23		LNG Plant	1047	I	[60]
23/12/2003	China Kaixian Chongqing	4000	243		Gas Well		I	[55]
21/09/2001	France Toulouse	3000	30		Fertiliser Plnt	1100	D	[60]
15/03/2001	Brazil Campos Basin		10	200	Oil Rig Expls	755	I	[60]
		*(1)*	*(2)*	*(3)*	*(4)*	*(5)*		*(6)*
**Period A**	**2001–2010**	**Nr**	**Inj. (2)**	**Fat. (1)**	**Evacuated**	**Ratio (2):(1)**		**Cost**
*(a)*	Total	18	8210	1512	523,600	5.4		14,035
*(b)*	Avg. per accident		456.1	84.0				780
*(c)*	Developed Countries	5	3310	120	100	27.5		12,109
*(d)*	Avg. per accident		662.0	24.0				2422
*(e)*	Industrialsg. Countries	13	4900	1392	523,500	3.5		1926
*(f)*	Avg. per accident		376.9	107.1				148

**Table 2:** Period B, 1991–2000.

**Date**	**Country/Location**	**Inj.**	**Fatal.**	**Evac.**	**Context**	**Cost**	**Type**	**Source**
10/07/2000	Nigeria Adeje Twn		250		Oil Pipeline		I	[60]
25/06/2000	Kuwait Mina Al-Ahmadi	50	5		Oil Refinery	634	I	[60]
13/05/2000	Netherlands Enschede	950	18		Explsvs Storg		D	[60]
17/10/1998	Nigeria Niger Delta		1078		Oil Pipeline	58	I	[61]
15/09/1997	India Visakhapatnam	100	60	60,000	LPG Tanks	24	I	[60]
21/11/1996	Perto Rico San Juan	100	33		Gas Pipeline	13	I	[61]
26/07/1996	Mexico Cactus	30	6		LPG Plant	217	I	[60]
28/04/1995	S. Korea Taegu	100	101	10,000	LPG Pipeline		D	[60]
07/12/1994	S. Korea Seoul	50	7	10,000	LNG Leak		D	[60]
26/11/1993	China Shuangpai		61		Explsvs Plant		I	[60]
02/11/1994	Egypt Donca		410		Oil Refinery		I	[60]
25/05/1993	Venezuela Lake Maracaibo		11		Propane Plant	178	I	[60]
28/09/1993	Venezuela Las Tejerias		36		Gas Pipeline		I	[60]
01/11/1993	Vietnam Nam Khe	50	47		Petrol Pipeline		I	[60]
06/08/1993	China Shenzhen	170	12		Warehouse Fire->Gas Plant		I	[60]
04/08/1993	Colombia Remeios		430		Oil Spillage		I	[60]
05/08/1993	China Zhengzhou	30	27		Chemicals Fire		I	[60]
11/01/1993	China Baohe		70		Gas Storg.		I	[55]
07/01/1993	S. Korea Chongju	50	27		LPG Fire		D	[60]
16/10/1992	Japan Sodegaura	10	10		Oil Refinery	287	D	[60]
01/09/1992	Greece Elefsina	20	20		LPG Pipeline		D	[60]
08/08/1992	Turkey Corlu	60	32		Methane Explsn		I	[60]
09/05/1992	Canada Plymouth Nova Scotia		26		Coal Mine/Gas	4	D	[61]
29/04/1992	India New Delhi	20	43		Chemicals Fire		I	[60]
22/04/1992	Mexico Guadalajara	1460	206		Petrol Pipeline	122	I	[60]
24/03/1992	Senegal Dakar	300	40		Ammonia Tank		I	[60]
24/09/1991	Thailand Bangkok		63		LPG Transport		I	[60]
01/09/1991	China Shaxi	350	30		Pesticide Fire		I	[60]
01/05/1991	USA Sterlington	120	8	500	Fertiliser Plant		D	[60]
		*(1)*	*(2)*	*(3)*	*(4)*	*(5)*		*(6)*
**Period B**	**2001–2010**	**Nr**	**Inj. (2)**	**Fat. (1)**	**Evacuated**	**Ratio (2):(1)**		**Cost**
*(a)*	Total	29	4020	3167	80,500	1.3		1537
*(b)*	Avg. per accident		138.6	109.2				53
*(c)*	Developed Countries	8	1300	217	20,500	6.0		291
*(d)*	Avg. per accident		162.5	27.1				36
*(e)*	Industrialising Countries	21	2720	2918	60,000	0.9		1246
*(f)*	Avg. per accident		129.5	139.0				59

**Table 3:** Period C, 1981–1990.

**Date**	**Country/Location**	**Inj.**	**Fatal.**	**Evac.**	**Cause**	**Cost**	**Type**	**Source**
15/11/1990	Portugal Porto de Leixoes	80	14		Propane Storg.	32	D	[60]
06/11/1990	India Nagothane	20	31		Gas Cracker	41	I	[60]
05/11/1990	India Maharashtra		35		Gas Cracker		I	[60]
23/10/1989	USA Pasadena	310	23	1300	Plastics Prd.	1275	D	[60]
04/06/1989	USSR Ufa Siberia	710	654	500	Gas Pipeline	17	I	[60,61]
20/03/1989	USSR Ionava	50	6	30,000	Fertiliser Plant		I	[60]
07/03/1989	Belgium Antwerp	10	32		Cycl.Ether Plant	145	D	[60]
11/11/1988	India Bombay	20	35		Oil Pipeline		I	[60]
06/07/1988	UK Piper Alpha		165		Oil Rig Expls.	1860	D	[60,61]
05/05/1988	U.S. Norco	50	7		Oil Refinery	493	D	[60]
22/01/1988	China Shanghai	20	25		Oil Refinery		I	[60]
12/12/1987	India Maharashtra	20	25		Naphta Pipeline		I	[60]
26/04/1986	Ukraine Chernobyl	600,000	4056	336,000	Nucl.Power Plant	7169	I	[60,61]
01/11/1985	India Padaval	80	43		Petrol Storg.		I	[60]
06/07/1985	USA Clinton	10	5		Ammonia Plant	24	D	[60]
19/05/1985	Italy Priolo	10	23		Ethylene Plant	136	D	[60]
03/12/1984	India Bhopal	500,000	20,000		Pesticide Plant	613	I	[60]
01/12/1984	Pakistan GahriDhoda		60		Gas Pipeline		I	[60]
19/11/1984	Mexico San Juan Ixhuatepec	6400	650		LPG Storg.	43	I	[60]
23/07/1984	USA Romeoville	80	15		Oil/Petrol Refnry	403	D	[60]
24/02/1984	Brazil Cubatao	220	508		Gas Piplne/Plant		I	[60]
29/09/1983	India Dhulwari	100	41		Petrol Storg.		I	[60]
31/08/1983	Brazil Pojuca	100	42	1000	Petrol Transp.		I	[60]
19/12/1982	Venezuela Tacoa	500	150	40,000	Oil Storg.	112	I	[60]
25/04/1982	Italy Todi	140	34		Gas Leak		D	[60]
15/02/1982	Canada Grd Banks		84		Oil Rig Sinkng		D	[60]
08/04/1981	Mexico Montanas	50	29		Transp. Chlorine		I	[60]
07/04/1981	USA Corpus Christi	30	9		Grain Storg.	67	D	[60]
		*(1)*	*(2)*	*(3)*	*(4)*	*(5)*		*(6)*
**Period C**	**1981–1990**	**Nr**	**Inj. (2)**	**Fat. (1)**	**Evacuated**	**Ratio (2):(1)**		**Cost**
*(a)*	Total	28	1,109,010	26,801	408,800	41.4		12,430
*(b)*	Avg. per accident		39,607.5	957.1				444
*(c)*	Develpd Countries	11	720	411	1300	1.7		4435
*(d)*	Avg. per accident		65.4	37.4				403
*(e)*	IndustrialsgCtrs	17	1,108,290	26,390	377,000	19.7		7995
*(f)*	Avg. per accident		65,193.5	1552.3				470

**Table 4:** Period D, 1971–1980.

**Date**	**Country/Location**	**Inj.**	**Fatal.**	**Evac.**	**Cause**	**Cost**	**Type**	**Source**
29/11/1980	Spain Ortuella		51		Propane Heater		D	[60]
21/10/1980	USA New Castle		5		Petrochem Plant	162	D	[60]
18/08/1980	Iran Gach Saran	50	80		Warehs Nitroglyc		I	[60]
16/08/1980	Ireland Bantry Bay		50		Oil Tanker Explsn	63	D	[60]
27/03/1980	UK Sector Ekofisk Field		123		Oil Rig Collapse	123	D	[61]
--/01/1980	USA Alaska		51		Oil Pump Station		D	[60]
03/06/1979	Thailand Phang Nga	20	50		Oil?		I	[60]
16/08/1980	Japan Shizuoka	220	15		Gas Pipeline		D	[60]
14/07/1978	Taiwan Kaoshiung	50	33		Plastics Prd.		D	[60]
30/05/1978	USA Texas City	10	7		Oil Refinery	176	D	[60]
11/02/1978	Mexico PobladoTres	30	40		Gas Pipeline		I	[60]
12/06/1978	Japan Sendai	350	21		Oil Storg.		D	[60]
10/12/1977	USA Westwego	10	35		Grain Storg.		D	[60]
10/12/1977	Colombia Pasacabolo	20	30		Fertiliser Plant		I	[60]
03/04/1977	Qatar Umm Said	60	7		Propane Storg Plt	262	I	[60]
09/03/1976	USA Oven Fork Kentucky		26		Coal Mine/Gas	9	D	[61]
--/12/1976	Colombia Carthagene	30	30		Fertiliser Plant		I	[60]
07/11/1975	Netherlands Beek	110	14		Naphta Cracker	89	D	[60]
17/08/1975	USA Philadelphia	20	8		Oil Refinery	49	D	[60]
08/08/1975	China Henan Province Dam		171,000		Dam Collapse	9310	I	[61]
10/02/1975	Belgium Antwerp	10	6		Plastics Prd.	134	D	[60]
03/11/1974	Japan Tokyo Bay		33		LPG/Naphta Tanker		D	[60]
19/06/1974	USA Decatur Illinois	360	7		Butane Tank Cars	72	D	[60]
01/06/1974	UK Flixborough	80	28		Chemical Plant	266	D	[60]
30/10/1978	Romania Pitesti		100		Petrochem Refinery		I	[60]
21/09/1972	Brazil Duque de Caxias	50	37		Refinery/LPG Plant		I	[60]
30/03/1972	Brazil Rio de Janeiro		40		Rfnry LPG Storg.		I	[60]
26/02/1972	USA Staten Island		40		LNG Storage	43	D	[60,61]
26/02/1972	USA Buffalo Creek	1100	125	4000	Coal Slurry Flood	81	D	[60]
--/--/1972	Japan Yokkaidi	980	76		Chemical Plant		D	[60]
		*(1)*	*(2)*	*(3)*	*(4)*	*(5)*		*(6)*
**Decade C**	**1981–1990**	**Nr**	**Inj. (2)**	**Fat. (1)**	**Evacuated**	**Ratio (2):(1)**		**Cost**
*(a)*	Total	30	3560	172,168	4000	0.02		10,839
*(b)*	Avg. per accident		118.7	5738.9				361
*(c)*	Developed Countries	20	3300	754	4000	6.9		1267
*(d)*	Avg. per accident		165.0	37.7				63
*(e)*	IndustrlsgCountrs	10	260	171,414		0.00016		9572
*(f)*	Avg. per accident		26.0	17,141.4				957
